# Evaluating Conversational Agents for Mental Health: Scoping Review of Outcomes and Outcome Measurement Instruments

**DOI:** 10.2196/44548

**Published:** 2023-04-19

**Authors:** Ahmad Ishqi Jabir, Laura Martinengo, Xiaowen Lin, John Torous, Mythily Subramaniam, Lorainne Tudor Car

**Affiliations:** 1 Lee Kong Chian School of Medicine Nanyang Technological University Singapore Singapore Singapore; 2 Future Health Technologies Singapore-ETH Centre Campus for Research Excellence And Technological Enterprise Singapore Singapore; 3 Beth Israel Deaconess Medical Center Boston, MA United States; 4 Institute of Mental Health Singapore Singapore; 5 Saw Swee Hock School of Public Health National University of Singapore Singapore Singapore; 6 Department of Primary Care and Public Health School of Public Health Imperial College London London United Kingdom

**Keywords:** conversational agent, chatbot, mental health, mHealth, mobile health, taxonomy, outcomes, core outcome set

## Abstract

**Background:**

Rapid proliferation of mental health interventions delivered through conversational agents (CAs) calls for high-quality evidence to support their implementation and adoption. Selecting appropriate outcomes, instruments for measuring outcomes, and assessment methods are crucial for ensuring that interventions are evaluated effectively and with a high level of quality.

**Objective:**

We aimed to identify the types of outcomes, outcome measurement instruments, and assessment methods used to assess the clinical, user experience, and technical outcomes in studies that evaluated the effectiveness of CA interventions for mental health.

**Methods:**

We undertook a scoping review of the relevant literature to review the types of outcomes, outcome measurement instruments, and assessment methods in studies that evaluated the effectiveness of CA interventions for mental health. We performed a comprehensive search of electronic databases, including PubMed, Cochrane Central Register of Controlled Trials, Embase (Ovid), PsychINFO, and Web of Science, as well as Google Scholar and Google. We included experimental studies evaluating CA mental health interventions. The screening and data extraction were performed independently by 2 review authors in parallel. Descriptive and thematic analyses of the findings were performed.

**Results:**

We included 32 studies that targeted the promotion of mental well-being (17/32, 53%) and the treatment and monitoring of mental health symptoms (21/32, 66%). The studies reported 203 outcome measurement instruments used to measure clinical outcomes (123/203, 60.6%), user experience outcomes (75/203, 36.9%), technical outcomes (2/203, 1.0%), and other outcomes (3/203, 1.5%). Most of the outcome measurement instruments were used in only 1 study (150/203, 73.9%) and were self-reported questionnaires (170/203, 83.7%), and most were delivered electronically via survey platforms (61/203, 30.0%). No validity evidence was cited for more than half of the outcome measurement instruments (107/203, 52.7%), which were largely created or adapted for the study in which they were used (95/107, 88.8%).

**Conclusions:**

The diversity of outcomes and the choice of outcome measurement instruments employed in studies on CAs for mental health point to the need for an established minimum core outcome set and greater use of validated instruments. Future studies should also capitalize on the affordances made available by CAs and smartphones to streamline the evaluation and reduce participants’ input burden inherent to self-reporting.

## Introduction

Recent technological advances have led to the proliferation of digital interventions, such as conversational agents (CAs), in different areas of health care, including mental health [1]. CAs, also known as chatbots, are multimodal systems that support conversational interactions with users through text, voice, and images [2]. CAs offer scalability and 24-hour availability, which allows timely interventions focusing on management, treatment, prevention of mental health conditions, and improvement of mental well-being. Woebot, for example, is a primarily text-based CA, which provides timely check-ins with users to encourage mood tracking and deliver general psychoeducation based on cognitive behavior therapy and behavior change tools [3,4]. A recent systematic review on the effectiveness of CA-delivered interventions for depression and anxiety showed a significant decrease in depressive symptoms in adults [5]. However, the low quality of overall evidence and limited well-designed randomized controlled trials (RCTs) [5,6] suggest the need to improve the quality of trials further.

Recent reviews on the use of CAs for mental health suggest heterogeneity of the outcome measurements used [6-9]. This issue is not limited to CAs but involves digital health interventions (DHIs) in general [10-12]. For example, studies that evaluated mental health DHIs typically reported user experience, satisfaction, and engagement with the intervention without clear and standardized criteria to evaluate them [6,8,11]. For instance, a study may report subjective feedback from users but not include objective measurements, such as average duration of use and the number of modules completed, to provide a better understanding of the context of use [11,13]. While efforts had been made to set a standardized benchmark for subjectively reported user experience outcomes [14], there are no gold standards that objectively measure these outcomes [8]. This is further hampered by (1) the lack of standardized taxonomy to describe the breadth of measurement instruments available [8,10,11,15] and (2) the use of outcome measurement instruments without validity evidence, which affects the credibility of study findings [16]. Lastly, objective measures to assess the performance of the system are also important [9]. This includes measures that track technical issues, such as system crashes and glitches, to understand if the CA is working well during the intervention. Similar to user experience, subjective measures of technical issues should be explored in conjunction with objective measures. This may include objective counts of error-handling messages sent in addition to user subjective experience of the dialogues or CAs in general [2].

The method of data collection is as important as the outcome measurement instrument used to improve the quality of clinical trials. Traditional methods via pen-and-paper and phone-based surveys can be costly and burdensome to participants and researchers alike [17]. Varied means of data collection approaches are particularly relevant in studies on digital mental health and well-being interventions, which are prone to high dropout rates [18]. This may include innovative ways of data collection, ranging from the integration of web-based survey platforms, such as Qualtrics and Google Form [4], in the system to the collection of passive smartphone sensor information in the form of digital biomarkers [19]. Passive and ongoing data collection also allows for more frequent measurements that can be used to reduce participants’ input burden [19]. To improve the transparency and quality of the evaluation and reporting of CA-delivered interventions focusing on mental health and well-being, there is a need to identify the choice of outcomes and outcome measurement instruments used in studies to date. Correspondingly, in this review, we aimed to (1) identify the types of outcome measurement instruments reported in studies assessing the effectiveness of mental health interventions delivered by CAs, (2) identify the data collection methods used (eg, pen-and-paper or technology-assisted methods) and the frequency of data collection in these studies, and (3) determine the prevalence of outcome measurement instruments with validity evidence employed in mental health interventions delivered by CAs.

## Methods

### Overview

This report follows the Joanna Briggs Institute scoping review guidelines [20] and the PRISMA-ScR (Preferred Reporting Items for Systematic Reviews and Meta-Analyses Extension for Scoping Reviews) [21] checklist (Multimedia Appendix 1). The protocol was registered on the Open Science Framework database (protocol ID: DEG4K).

### Search Strategy

A search strategy including 63 terms that define or are synonymous with CAs was designed and used in a series of scoping reviews to explore the use of CAs in health care (Multimedia Appendix 2). The search included sources of peer-reviewed research specializing in medical, psychology, engineering, multidisciplinary, and grey literature. The search was performed on April 26, 2021, in the PubMed, Cochrane Central Register of Controlled Trials, PsychINFO, Web of Science, and Embase (Ovid) databases and in the first 10 pages of Google Scholar and Google [22]. These databases were chosen based on our experience in developing similar reviews on CAs in health care and were optimized by a medical librarian for this review [22].

### Eligibility Criteria

This scoping review included experimental primary studies, such as RCTs, cluster randomized trials, quasirandomized trials, controlled before-and-after studies, uncontrolled before-and-after studies, interrupted time series, pilot studies, and feasibility studies. Nonexperimental studies, such as observational studies, reviews, qualitative studies, editorials, personal communications, conference abstracts, and articles where the full text was not available, were excluded. We included mental health interventions delivered by CAs, including the promotion of mental well-being, and the prevention and management of mental health disorders, including but not limited to mood disorders, psychosis, posttraumatic stress disorder, and substance use disorders. We excluded studies that focused primarily on comparing or evaluating specific CA features, those that did not report health outcomes, and those whose dialogue was derived from human operators (“Wizard of Oz”).

Within the context of this study, a CA was defined as a human-machine interface that holds human-like synchronous conversation via text, voice, images, video, or multimodal outputs, and autonomously interprets user input via decision trees or complex neural network algorithms [22]. CAs could be preconfigured with a set of predefined responses (rule-based CAs) or enhanced with natural language processing or machine learning (artificial intelligence [AI]-enhanced CAs) [22]. An embodied CA was defined as a CA that includes an avatar with human-like features, which can mimic human movements and facial expressions [22].

### Screening, Data Extraction, and Analysis

The title and abstract screening was performed by 2 reviewers (AIJ and XL) independently and in parallel on Covidence [23]. Studies included in this step were uploaded to EndNote X9 (Clarivate) for full-text review, which was performed in parallel by AIJ and XL. Discrepancies among the reviewers were settled via discussions between the reviewers or with input from a third reviewer (LM). The data extraction form was developed by the research team using Microsoft Excel (Microsoft Corp). The data extraction was performed in parallel by AIJ and XL. The form was piloted on 3 studies and then amended based on feedback to better fit the research aims. The extracted data were compared, and disagreements were resolved via discussion or input from LM acting as the arbiter. Data were presented in a diagrammatic or tabular form accompanied by a narrative summary of the findings.

The outcomes were categorized into clinical, technical, and user experience outcomes. Clinical outcomes were defined as “measurable changes in health, function, or quality of life” [22]. These outcomes derive directly or indirectly from the expected mechanisms of the CA-delivered intervention. Clinical outcomes were categorized based on the Core Outcome Measures in Effectiveness Trials (COMET) Initiative’s medical research outcome taxonomy comprising 38 categories such as “21: Psychiatric Outcomes,” “26: Physical Functioning,” and “28: Emotional Functioning” (see Multimedia Appendix 3 for the definitions) [15]. User experience outcomes “encompassed all direct and indirect experiences of the user while interacting with the CA” [22]. These included the subjective self-reported experience of the intervention, such as system usability, satisfaction with the CA, and interviews with users. We also included objective engagement measures, which were further categorized based on a previous systematic review of mobile health (mHealth) interventions for depression [10]. Technical outcomes were measures used to evaluate the performance of the CA itself related to its technical interface, system crashes, and dialogue system, such as chatbot response generation [9]. Unlike previous research [9], we did not consider users’ experiences of glitches and errors as technical outcomes. Rather, technical outcomes strictly referred to objective measures of system performance, such as the number of errors from the system log.

The outcome measurement instruments were categorized into those measuring outcomes objectively and subjectively. Objective measures included (1) “sensor data” to monitor human behavior or physiological changes using either external sensors, such as respiratory sensors for breathing rate [24], or smartphone “passive sensing modules,” such as gyroscopes, GPS modules, or accelerators [19]; and (2) “objective engagement measures” defined as data captured passively by the system log while the user interacts with the system [8,10]. Subjective measures included those measured with instruments or tools (eg, questionnaires) that involve self-reporting by the participant using either pen-and-paper or digital means. These measurement instruments may assess health-related outcomes, such as symptoms, symptom burden, health-related quality of life, usability, or satisfaction with the system.

Validity evidence of the outcome measurement instruments was extracted based on the COMET Initiative’s taxonomy of measurement properties [25], comprising 3 quality domains: reliability, validity, and responsiveness. The reliability domain includes internal consistency of the items, reliability, and measurement errors not attributed to true changes in the construct measures. The validity domain includes the content, construct, and criterion validity of the instruments. The responsiveness domain covers longitudinal validity or the ability of the instrument to detect change over time. The validity evidence was extracted based on the measurement properties that were reported directly from the studies or referenced by the studies. We recorded the relevant details from the other references, including if the study reported more than one reference.

## Results

### Overview

**Figure 1:**
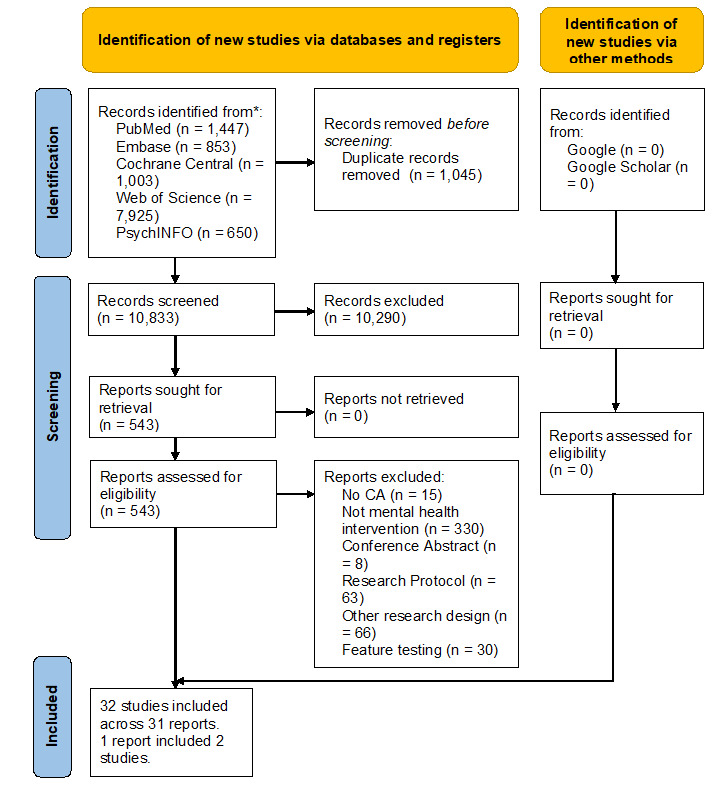
PRISMA (Preferred Reporting Items for Systematic Reviews and Meta-Analyses) flowchart. CA: conversational agent.

The search strategy retrieved 10,833 papers after removing duplicates, of which 543 were eligible for full-text screening, and 31 papers were included. We reported a total of 32 studies as 1 paper included 2 studies. Figure 1 presents the study selection process.

### Characteristics of the Included Studies

Of the 32 studies included in this review, 25 (78%) [3,4,13,24,26-45] were published in the past 5 years, with 5 studies [4,13,26,34,35] published in the first quarter of 2021 (Table 1). Most of the studies (16/32, 50%) were from the United States of America [3,4,24,30,32-34,42-49].

**Table 1:** Characteristics of the included studies.

Study characteristic		Value (N=32), n (%)
**Year of publication**		
	<2017	7 (22)
	≥2017	25 (78)
**Country**		
	United States of America	16 (50)
	United Kingdom	7 (22)
	Germany	1 (3)
	Italy	1 (3)
	Sweden	1 (3)
	Japan	3 (9)
	Korea	2 (6)
	China	1 (3)
**Type of study design**		
	RCT^a^	13 (41)
	Pilot study	8 (25)
	Before-and-after study	6 (19)
	Feasibility study	3 (9)
	Nonrandomized comparison	1 (3)
	Crossover RCT	1 (3)
**Study duration**		
	<8 weeks	25 (78)
	≥8 weeks	7 (22)
**Sample population**		
	Healthy adults	23 (72)
	Inpatients/outpatients	9 (28)
**Target clinical outcome**		
	Treatment and monitoring	21 (66)
	Education and training	11 (34)
**Target disorder/intervention**		
	Mental well-being	17 (53)
	Co-occurring depression and anxiety	4 (13)
	Depression only	3 (9)
	Others^b^	8 (25)

^a^RCT: randomized controlled trial.

^b^Height phobia, panic disorder, anxiety only, suicide prevention, gambling disorder, attention-deficit/hyperactivity disorder, irritable bowel syndrome, and substance abuse.

Most studies (22/32, 69%) included at least one comparison group [3,27-39,41-46,50,51]. An RCT design was used in 13 studies (43%) [3,27,28,30-32,37,38,41,42,44,45,50]. Moreover, 6 studies (19%) reported before-and-after trials with no comparison group [4,13,26,47,52]. Most of the studies (23/32, 72%) were conducted on healthy adults [3,4,13,24,26-28,30-32,36,38-43,45,46,48,51,52].

The included studies were primarily focused on promoting mental well-being (17/32, 53%) by offering education and training through psychoeducation, cognitive, or behavioral training, such as mindfulness exercises (6/17, 35%) [24,33,36,42,46,48], or monitoring of well-being indicators, such as daily emotions (11/17, 65%) [13,26-28,30,39-41,45,51,52]. Fifteen studies assessed the treatment of specific mental health disorders. Among these, 3 assessed depression only [44,49,50], 1 assessed anxiety only [29], and 4 assessed co-occurring depression and anxiety [3,32,43].

Among 21 studies that included a comparison group, most (14/21, 67%) reported that the CAs were more effective than the comparison approach [3,30-32,34-36,39,41,44,46,48-50], 4 (19%) reported mixed findings [33,37,43,51], and 5 (24%) reported no difference between the groups [27,28,38,42,45]. In studies that lasted more than a day but less than 8 weeks and provided attrition data (20/32, 69%), the average attrition rate was 19.16% (range 0%-56%) [3,4,13,31-40,43,45-47,50-52]. Three studies that lasted 4-8 weeks reported an attrition rate of 0% [13,32,52]. Two studies that lasted more than 8 weeks reported attrition rates of 35% [49] and 9% [44]. Multimedia Appendix 4 presents a detailed summary of the included studies.

Most CAs were deployed on a web-based application (7/32, 22%) [27,28,30,39,46,51,52], a standalone smartphone app (7/32, 22%) [4,13,29,34-37], or a laptop/desktop-based program (7/32, 17%) [26,31,43,47,48,50]. Among the 32 studies, 14 (44%) included embodied CAs [24,29-31,39,43-50], 11 (34%) included CAs characterized by avatars [3,4,13,32-35,37,40-42], and 7 (22%) did not specify the type of CA visualization [26-28,36,38,51,52]. Moreover, there were 18 (56%) rule-based CAs [3,24,26,30,33,34,36-38,41,42,44-50] and 14 (44%) AI-enhanced CAs [4,13,27-29,31,32,35,39,40,43,51,52]. The CAs were mostly coach-like (23/32, 72%) [3,4,13,24,29-38,40,43-49], characterized by encouraging, motivating, and nurturing personalities (Table 2). Among the 32 studies, 6 (19%) CAs were presented as a health care professional [26-28,41,50,51], 2 (6%) used informal language and conversed with users like a friend [42,52], and 1 (3%) [39] showed a knowledgeable personality based on content created and informed by medical experts [22].

**Table 2:** Conversational agent characteristics.

CA^a^ characteristic			Value (N=32), n (%)
**Type of CA**			
	ECA^b^	14 (44)	
	Avatar only	11 (34)	
	Not specified	7 (22)	
**Delivery channel**			
	Web-based application	7 (22)	
	Standalone smartphone app	7 (22)	
	Computer/laptop-based program	7 (22)	
	Messaging app–based approach^c^	6 (19)	
	Tablet computer	4 (13)	
	Hybrid approach^d^	1 (4)	
**Type of CA by dialogue modality**			
	Rule-based CA	18 (56)	
	AI^e^-enhanced CA	14 (44)	
**CA personality**			
	Coach-like personality	23 (72)	
	Health care professional–like personality	6 (19)	
	Informal-like personality	2 (6)	
	Knowledgeable personality	1 (3)	

^a^CA: conversational agent.

^b^ECA: embodied conversational agent.

^c^Facebook, Slack, Telegram, or LINE.

^d^Both standalone and web-based applications.

^e^AI: artificial intelligence.

### Types of Outcome Measurement Instruments

In total, there were 203 outcome measurement instruments, of which 149 were used in 1 study only. Sixteen instruments were included more than once using the same version, a translated version, or a shortened version of the instruments (Multimedia Appendix 5). Three of these instruments were reported in 3 separate studies involving the same CA, that is, MYLO [27,28,51].

**Figure 2:**
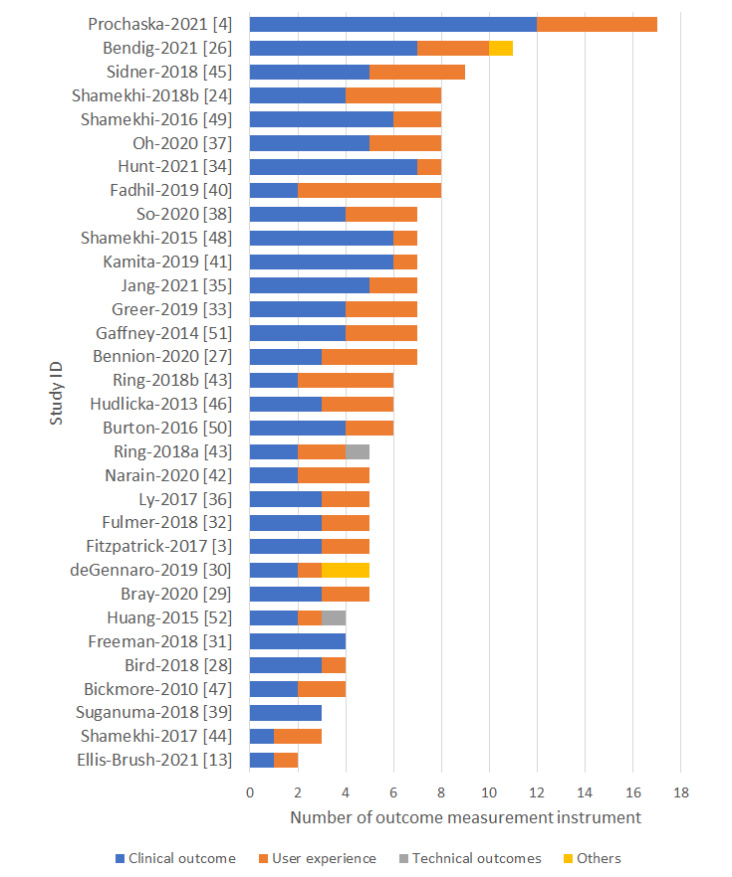
Types of outcomes by the study ID.

All the studies included at least one clinical and one user experience outcome measurement instrument, except for 2 studies that only measured clinical outcomes [31,39] (Figure 2). Most of the outcome measurement instruments (123/203, 60.6%) measured clinical outcomes, 36.9% (75/203) measured user experience outcomes, and 1.0% (2/203) included technical outcomes measuring the accuracy of the stress feature detection [52] and the accuracy of the emphatic feedback function [43]. Moreover, 3 (1.5%) outcome measurement instruments were categorized as *others* as they measured the effectiveness of the experiment manipulation unrelated to clinical, user experience, or technical outcomes, such as whether the user paid attention to the experiment manipulation information [30]. Figure 2 describes the numbers and types of outcomes measured by the included studies.

Table 3 maps all the outcome measurement instruments according to clinical, user experience, and technical outcome categories. Based on the COMET Initiative’s clinical outcome taxonomy, most of the clinical outcome measurement instruments measured psychiatric outcomes (57/123, 46.3%), followed by emotional functioning/well-being outcomes (31/123, 25.2%) and cognitive functioning outcomes (21/123, 17.1%). Two studies used external sensors to objectively measure physiological changes, specifically the galvanic skin response, to assess physiological arousal [48] and breathing rate [24]. Most of the instruments measuring clinical outcomes (92/123, 74.8%) were based on published literature. A quarter of the studies (31/123, 25.2%) used original tools, and these mostly used 1-item questionnaires to assess emotional functioning/well-being outcomes (12/31, 39%).

**Table 3:** Frequencies of the categories and subcategories of all outcome measurement instruments.

Categories^a^ and subcategories				Value (N=203), n (%^b^)
**Clinical outcomes^c^**				
	Psychiatric outcomes			57 (28.1)
	Emotional functioning/well-being			32 (15.8)
	Cognitive functioning			21 (10.3)
	Social functioning			4 (2.0)
	Adverse events			4 (2.0)
	Delivery of care			2 (1.0)
	Physiological data			2 (1.0)
	Physical functioning			1 (0.5)
	Gastrointestinal outcomes			1 (0.5)
**User experience outcomes**				
	**Subjective user experience outcomes**			
		User experience with the overall system	34 (16.7)	
		User experience with the CA^d^	15 (7.4)	
		User attitudes toward technology	6 (3.0)	
	**Objective user engagement measurement^e^**			
		Total duration of use	10 (4.9)	
		Interaction with the CA	8 (3.9)	
		Assessment of active use	7 (3.4)	
		Total number of sessions	5 (2.4)	
		Use of specific program features	4 (2.0)	
		Average duration of the session	4 (2.0)	
		Completion of a structured module	2 (1.0)	
		Program use by day or week	2 (1.0)	
		Adherence to usage instructions	1 (0.5)	
**Technical outcomes**				
	Accuracy of the NLP^f^ classifier			2 (1.0)
**Other outcomes**				
	Experimental manipulation tests			3 (1.5)

^a^Categories are not mutually exclusive.

^b^The percentages do not add to 100% as some outcomes are mapped to two or more subcategories.

^c^Subcategories are based on the core outcome set taxonomy of clinical outcomes.

^d^CA: conversational agent.

^e^Subcategories are based on a systematic review [10] of engagement with a mobile health intervention for depression.

^f^NLP: natural language processing.

Among the user experience outcomes, the instruments were grouped into 2 major categories: subjective measures of user experience (54/75, 72%) and objective engagement measures via system log data (20/75, 27%). Thirty studies reported subjective measures of user experience*.* The majority (43/54, 80%) included questionnaires developed by the researchers to measure various aspects of user experience, and 12 studies (12/54, 22%) used validated or previously published instruments. Most studies (34/54, 63%) measured users’ experiences of the whole system using validated questionnaires such as the System Usability Scale (SUS) [53]. Almost a third of the studies (15/54, 28%) explored user experience and satisfaction with the CA, including satisfaction and likability of the CA or the user-CA working alliance [54]. Other studies (6/54, 11%) included a questionnaire on users’ attitudes toward the technology.

Among the 32 studies, 20 (63%) reported one or more objective engagement measures to collectively describe user engagement with the CA. Multimedia Appendix 6 details the various definitions for the system log data collected by the studies. Most studies (14/20, 70%) reported the total duration of use in general; however, some studies (7/20, 35%) included a specific definition of “active use.” This included completing specific tasks, such as at least two user responses via the conversation interface within 5 minutes [33], or completing specific tasks within a session [3,4,35,36,47,50]. Other studies defined engagement based on the number of interactions with the CA [4,32,38,52].

### Data Collection Method

Overall, most of the outcomes (170/203, 83.7%) were collected via self-reported questionnaires, with an average of 5 self-reported instruments per study (range 2-16). Almost all the clinical outcomes were collected via self-reported questionnaires (120/123, 97.6%). Three studies measured clinical outcomes using nonquestionnaire-based instruments, including qualitative analysis of participants’ conversation logs [51] or external physiological sensors [24,48]. Similarly, most of the user experience outcomes were collected via self-reported questionnaires (47/75, 63%), followed by objective engagement measures (22/75, 28%) and qualitative interview data (7/75, 9%). No study reported any outcomes using a passive sensing module via a smartphone.

**Figure 3:**
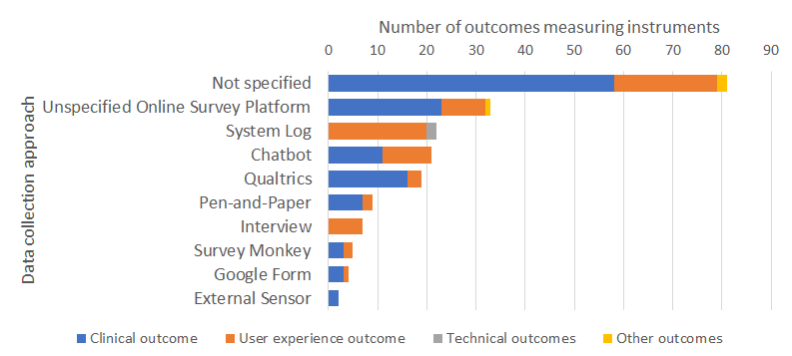
Data collection method employed in studies assessing conversational agent interventions in mental health.

One-third of the outcomes (61/203, 30.0%) were collected using a survey platform such as Qualtrics (19/203, 9.4%) (Figure 3). One-tenth of the studies collected data via system logs (22/203, 10.8%) or directly via the CA (21/203, 10.3%). Most studies (81/203, 39.9%), however, did not report the data collection platform.

### Types of Outcomes and Measurement Time Points

**Figure 4:**
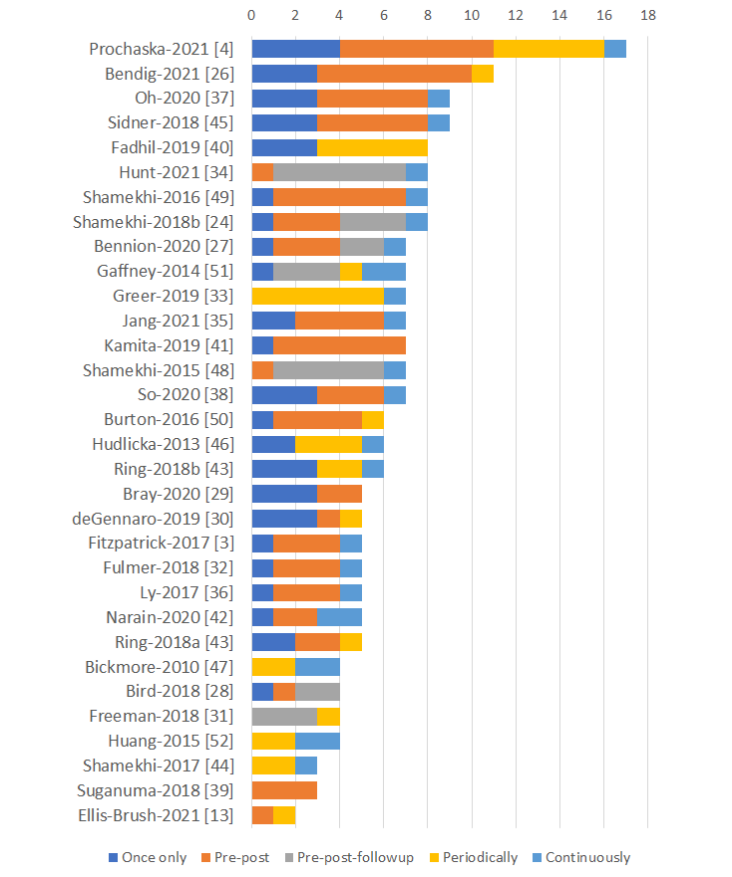
Frequency of data collection for various measurement time points. Periodically refers to daily, weekly, or posttask data collection. Continuously refers to data collection via sensor or system log data.

Most of the clinical outcomes were measured twice, that is, at baseline and postintervention (70/123, 56.9%), followed by thrice, that is, at baseline, postintervention, and during the follow-up period typically 2 to 12 weeks after the intervention (20/123, 16.3%). A minority of the clinical outcomes were measured periodically several times during the intervention either daily (4/123, 3.3%) or weekly (7/123, 5.7%). Ecological momentary assessments were used after a specific task to measure the interventions’ impact on users’ mood [4,26] or to assess adherence to medication or tasks [47]. Two studies included continuous data collection via external physiological sensors [24,48]. Most user experience outcomes were measured once postintervention (33/75, 44%) or continuously during the study using system log data (20/75, 27%). Moreover, 10 (14%) studies used ecological momentary assessments after specific tasks to collect user experiences, typically after a session with the CA (Figure 4).

### Validity Evidence

Validity evidence of the outcome measurement instruments was reported in less than half of the included studies (96/203, 47.3%). Among those without validity evidence, 95 (95/107, 88.8%) were researcher-designed instruments created or adapted for the study. Most clinical outcomes (83/123, 67.5%) included validity evidence, but only 12 user experience outcomes (12/75, 16%) included or cited validity evidence. One of the technical outcomes included validity evidence of the stress detection module [52]. Among the instruments that reported at least one validity evidence, most described or cited reliability statistics (82/96, 85%) or concurrent, convergent, discriminant, or construct validity statistics (91/96, 95%). A minority of the studies (8/97, 8%) cited or included responsiveness or sensitivity statistics.

### Association Between Types of Outcomes

One-fifth of the studies (8/32, 25%) analyzed clinical outcomes in association with user experience outcomes [4,13,27,35,37,40,43,51]. Four studies (4/8, 50%) compared psychological symptom outcomes and various user experience outcomes, including SUS, working alliance with CAs, satisfaction, and objective engagement measurements such as duration of use [4,35,37,43]. Three studies (3/8, 38%) compared cognitive functioning, specifically self-efficacy, with user experience [4,13,40]. Two studies (2/8, 25%) compared emotional functioning/well-being and user experience outcomes [27,51]. User experiences were associated with better clinical outcomes in 4 of the studies [27,35,43,51]. Moreover, 3 studies did not find any associations between the 2 types of outcomes [13,37,40], and 1 found that better user experience was related to a reduction in some clinical outcomes but no association with others [4]. All technical outcomes were reported separately.

## Discussion

### Principal Findings

In this scoping review, we assessed 32 studies reporting outcome measurement instruments used to evaluate CA-delivered mental health interventions, their context of use, the method and frequency of data collection approaches, and the prevalence of validity evidence for the outcome measurement instruments. We identified 203 outcome measurement instruments, out of which 150 were unique instruments that were created or adapted specifically for the study. Most instruments measured clinical outcomes and used self-reported instruments. More than half of the instruments did not report validity evidence. In studies that reported the outcome instruments’ validity evidence, reliability and construct validity were the most reported.

### Comparison With Prior Work

Our review found heterogeneity in the choice of outcome measurement instruments used. This is similar to other reviews on this topic focusing on CA-delivered mental health interventions [7-9]. A recent review of commonly used measures to assess system usability and user experience further noted a lack of a unified measurement instrument that measures all aspects of usability [55]. Systematic reviews on user adherence and engagement to DHIs also strongly suggested a more unified operationalization of engagement measures and their relationship with other subjectively reported outcomes [10,56]. These results point to a need for a core outcome set specific to understanding and quantifying user experience, usage, and adherence to CAs in mental health. For instance, studies should minimally include SUS or other validated usability questionnaires tailored for CAs [57] as measures of general usability for comparison among different CA systems [58]. This is in line with a recent meta-analysis that suggested the possibility of using the SUS score to benchmark usability across all digital mental health apps [14].

In our review, few studies assessed the relationship between clinical outcomes and other outcomes. A similar review evaluated the engagement with mHealth interventions for depression and found that fewer than half of the reviewed studies assessed the relationship between objective and subjective user experience and clinical outcomes [10]. The relationship between clinical and user experience outcomes is of particular interest in digital mental health interventions. Preliminary evidence suggests an association between better user engagement with the intervention website and a greater reduction in depression and anxiety symptoms [59]. A review has further suggested that a stronger therapeutic alliance with a digital mental health intervention may have an indirect relationship with clinical outcomes [59]. Therapeutic alliance, defined as the therapeutic relationship between the patient and therapist, is fundamental to the success of face-to-face psychological therapy [59,60]. Early evidence suggests that users may develop a therapeutic alliance with the CA [61]. However, factors, such as the quality of the app and user satisfaction, may affect the bond, although the evidence is still limited [62]. Our review, for example, suggested that higher objectively and subjectively measured user experiences with the CA platform were associated with better clinical outcomes in some studies [4,27,35,43,51], but a small number did not find any associations [4,13,37,40]. Future studies should further explore the relationship between clinical and nonclinical outcomes to understand the factors affecting the efficacy of CA-delivered mental health interventions.

Our review also found that most of the outcomes were self-reported electronically via unspecified survey platforms, which may suggest an underutilization of the technological affordances within the digital space. When objectively measured data were reported, they were typically used to understand user engagement with the application, via system log data. A recent review of wearables and smartphone-based passive sensing devices for mental health monitoring suggested innovative ways to incorporate inbuilt smartphone sensors to monitor general well-being and the symptoms of bipolar disorder and depression [19]. These appear to be necessary as half of our included studies measured more than five outcomes using self-reported questionnaires, which may increase participants’ input burden over time [63]. Shamekhi and Bickmore [24], for instance, found that the use of passive sensors provided a better user experience compared to having no sensor in a CA-led meditation session. Studies also typically collected self-reported data externally via survey platforms, such as Qualtrics and Survey Monkey, rather than collecting the data directly via the CA. A recent study suggested that a conversational survey collected directly by a CA is a reliable alternative to traditional surveys and may lead to improved response quality [64]. Our findings thus suggest the underutilization of the technological affordances made available by CA systems in terms of the data collection methodology.

Interestingly, our review identified only 2 studies that reported technical outcomes specifically related to the accuracy of the emotion detection modules in their ability to respond to user responses. While one study reported on technical glitches [3], this was mainly in the context of user experience and not for the entire performance of the CA during the study. These technical outcomes might be reported elsewhere as evidenced by a recent review on the complexity of technical outcomes in CA-delivered interventions for mental health [9]. However, without technical outcomes, it is difficult to fully evaluate the effectiveness of CAs to better understand the various ways that users interact with CAs.

Lastly, our review found that mental health CAs were mostly more effective than the comparison group in the included studies. In addition, the overall mean attrition rate was relatively lower than in other DHI studies [65]. This result is supported by a recent meta-analysis of 11 trials of CA-delivered psychotherapy, which showed significantly improved depressive symptoms among adults [5]. Another meta-analysis of smartphone-delivered mental health interventions further found that the mean study attrition for short-term studies was about 35.5% (95% CI 26.7-45.3) [65], which is higher than the average attrition rate of 19% found in our review and a recently published meta-analysis of CAs for depression and anxiety [5]. The systematic review acknowledged that the findings were still preliminary due to the limitations of the included studies. We hope that future studies will benefit from the recommendations provided in our scoping review by improving the overall quality of evaluation of mental health CAs.

### Strengths and Limitations

This scoping review has several strengths. First, we conducted a comprehensive literature search of multiple databases and grey literature sources. We prioritized the sensitivity of our search terms to capture the various representations of CAs used in mental health. Second, unlike other reviews in this area [66,67], our study analyzed all the outcomes included in CA-delivered interventions and provided a more granular mapping of these outcomes. This study, therefore, showcased the possible taxonomy of the outcomes measured in CA-delivered interventions that can be referenced by other researchers in this field.

Our study however has some limitations. First, given the novelty and multidisciplinary nature of the field, some unpublished literature presented at niche conferences and meetings may have been omitted. Second, during the data extraction process, we identified the validity evidence of the employed outcome measurement instruments based on the validation assessments cited in the included studies. Hence, the validity evidence captured here will more accurately reflect the reporting convention but not the actual validity of the instruments included. Some of the outcome measurement instruments used may have the necessary validity evidence but were not cited or reported by the included studies. Third, we used broad inclusion criteria that included studies using less robust experimental designs to provide a snapshot of the current state of the assessment for CA-delivered interventions in mental health.

### Recommendations for Future Research

The results from our scoping review suggested the need to standardize the outcome measurement instruments used in CAs for health care and specifically mental health. This may be done via a Delphi study or via existing guidelines [68] to establish the core outcome set directly related to CA functionalities, such as the definition of meaningful engagement with the CA, or determine user attitudes and perceptions toward the CA. This is necessary as engagement and the working alliance with the CA impact the way users interact with it [69]. Our results also suggested the need to include technical outcomes, such as system crashes, out-of-scope questions, and glitches in the dialogues during the implementation. This is to better understand the relationship among the technical issues faced, user experience, and clinical outcomes. Our results showed that most studies did not report the data collection method. We recommend including the data collection method, such as including the online survey platform name, or data collection via embedded programs within the intervention. This may inform future researchers to consider the effectiveness of various evaluation platforms for future interventions. Lastly, researchers may benefit from existing frameworks to guide the incorporation of passive sensing using smartphones or wearables for mental health interventions [70] to better use the technological affordances of CAs.

### Conclusion

This review suggests that studies on CA-delivered mental health interventions include a diverse set of clinical, user experience, or user engagement outcomes. Most of the measured outcomes were clinical outcomes, assessed electronically via an unspecified survey platform with uniquely created or adapted measurement instruments that lacked any reference to validity evidence. There is a need for a more consistent approach to the evaluation of these interventions, for example, through the development of guidelines with relevant experts and stakeholders. The review also suggested a greater need to capitalize on the affordances made available by CA systems and smartphones, such as passive sensing modules and conversation-based assessments, to streamline the assessment and reduce participants’ input burden when using self-reported instruments.

## Appendix

Multimedia Appendix 1 PRISMA-ScR (Preferred Reporting Items for Systematic Reviews and Meta-Analyses Extension for Scoping Reviews) checklist.

Multimedia Appendix 2 Search strategies.

Multimedia Appendix 3 Selected definitions for Core Outcome Measures in Effectiveness Trials (COMET)’s medical research outcome taxonomy.

Multimedia Appendix 4 Characteristics of the included studies.

Multimedia Appendix 5 Outcome measures included in more than one study.

Multimedia Appendix 6 System log data and definitions provided by the studies.

## Abbreviations

AI: artificial intelligence
CA: conversational agent
COMET: Core Outcome Measures in Effectiveness Trials
DHI: digital health intervention
mHealth: mobile health
PRISMA-ScR: Preferred Reporting Items for Systematic Reviews and Meta-Analyses Extension for Scoping Reviews
RCT: randomized controlled trial
SUS: System Usability Scale

## Data Availability

This scoping review does not contain primary data. The data set analyzed during this study is available from the corresponding author on reasonable request.
