# Supplementary material for: Evaluating Conversational Agents for Mental Health: Scoping Review of Outcomes and Outcome Measurement Instruments
Source: J Med Internet Res. 2023 Apr 19;25:e44548. doi: 10.2196/44548 (PMC10157460; doi:10.2196/44548)
Supplement: Multimedia Appendix 2 [file jmir_v25i1e44548_app2.docx]

**Multimedia Appendix 2.** Search strategies.

1. Artificially intelligent chatbot[Title/Abstract] OR Artificially intelligent chat agent[Title/Abstract] OR Automated virtual agent[Title/Abstract] OR Automated chat agent[Title/Abstract] OR Artificial intelligence chatbot[Title/Abstract] OR Artificial conversational entity[Title/Abstract] OR AI agent[Title/Abstract]

2. Chatterbox[Title/Abstract] OR Chatbot[Title/Abstract] OR Chatterbot[Title/Abstract] OR Chat assistant[Title/Abstract] OR chat bot[Title/Abstract] OR chat-bot[Title/Abstract] OR chatter bot[Title/Abstract]

3. Conversational agent[Title/Abstract] OR Conversational system[Title/Abstract] OR Conversational assistant[Title/Abstract] OR Conversational User Interface[Title/Abstract] OR Conversational Personal Assistant[Title/Abstract] OR Conversational interface[Title/Abstract] OR Conversational avatar[Title/Abstract] OR Conversational computer[Title/Abstract] OR Conversational humanoid[Title/Abstract] OR Conversational Character[Title/Abstract] OR Conversational bot[Title/Abstract] OR Conversational AI[Title/Abstract]

4. Dialog system[Title/Abstract] OR Dialogue system[Title/Abstract]

5. Embodied agent[Title/Abstract] OR Embodied conversational agent[Title/Abstract]

6. Interactive online character[Title/Abstract] OR Interactive virtual agent[Title/Abstract] OR Interactive agent[Title/Abstract] OR Interactive conversational assistant[Title/Abstract]

7. Intelligent virtual agent[Title/Abstract] OR Intelligent virtual assistant[Title/Abstract] OR Intelligent conversational assistant[Title/Abstract] OR Intelligent conversational avatar[Title/Abstract] OR Intelligent agent[Title/Abstract] OR Intelligent assistant[Title/Abstract]

8. Smartbot[Title/Abstract] OR Smart bot[Title/Abstract] OR Smart virtual assistant[Title/Abstract]

9. Online chat agent[Title/Abstract] OR Sociable agent[Title/Abstract] OR Relational agent[Title/Abstract]

10. Talk bot[Title/Abstract] OR Talking avatar[Title/Abstract]

11. Text-based healthcare chatbot[Title/Abstract] OR Text based dialogue system[Title/Abstract] OR Text-based synchronous chat[Title/Abstract] OR Speech recognition software[Title/Abstract] OR Voice recognition software[Title/Abstract]

12. Virtual assistant[Title/Abstract] OR Virtual agent[Title/Abstract] OR Virtual advisor[Title/Abstract] OR Virtual conversational agent[Title/Abstract] OR Virtual coach[Title/Abstract] OR Virtual online assistant[Title/Abstract] OR Virtual personal assistant[Title/Abstract]

13. #1 OR #2 OR #3 OR #4 OR #5 OR #6 OR #7 OR #8 OR #9 OR #10 OR #11 OR #12

**Search Strategy on CENTRAL**

#1 (Artificially intelligent chatbot OR Artificially intelligent chat agent OR Automated virtual agent OR Automated chat agent OR Artificial intelligence chatbot OR Artificial conversational entity OR AI agent):ti,ab,kw

#2 (Chatterbox OR Chatbot OR Chatterbot OR Chat assistant OR chat bot OR chat-bot OR chatter bot):ti,ab,kw

#3 (Conversational agent OR Conversational system OR Conversational assistant OR Conversational User Interface OR Conversational Personal Assistant OR Conversational interface OR Conversational avatar OR Conversational computer OR Conversational humanoid OR Conversational Character OR Conversational bot OR Conversational AI):ti,ab,kw

#4 (Dialog system OR Dialogue system):ti,ab,kw

#5 (Embodied agent OR Embodied conversational agent):ti,ab,kw

#6 (Interactive online character OR Interactive virtual agent OR Interactive agent OR Interactive conversational assistant OR Intelligent virtual agent OR Intelligent virtual assistant OR Intelligent conversational assistant OR Intelligent conversational avatar OR Intelligent agent OR Intelligent assistant):ti,ab,kw

#7 (Smartbot OR Smart bot OR Smart virtual assistant):ti,ab,kw

#8 (Online chat agent OR Sociable agent OR Relational agent):ti,ab,kw

#9 (Talk bot OR Talking avatar):ti,ab,kw

#10 (Text-based healthcare chatbot OR Text based dialogue system OR Text-based synchronous chat OR Speech recognition software OR Voice recognition software):ti,ab,kw

**Search Strategy on EMBASE**

1 (Artificially intelligent chatbot or Artificially intelligent chat agent or Automated virtual agent or Automated chat agent or Artificial intelligence chatbot or Artificial conversational entity or AI agent).ab,ti.

2 (Chatterbox or Chatbot or Chatterbot or Chat assistant or chat bot or chat-bot or chatter bot).ab,ti.

3 (Conversational agent or Conversational system or Conversational assistant or Conversational User Interface or Conversational Personal Assistant or Conversational interface or Conversational avatar or Conversational computer or Conversational humanoid or Conversational Character or Conversational bot or Conversational AI).ab,ti.

4 (Dialog system or Dialogue system).ab,ti.

5 (Embodied agent or Embodied conversational agent).ab,ti.

6 (Interactive online character or Interactive virtual agent or Interactive agent or Interactive conversational assistant).ab,ti.

7 (Intelligent virtual agent or Intelligent virtual assistant or Intelligent conversational assistant or Intelligent conversational avatar or Intelligent agent or Intelligent assistant).ab,ti.

8 (Smartbot or Smart bot or Smart virtual assistant).ab,ti.

9 (Online chat agent or Sociable agent or Relational agent).ab,ti.

10 (Talk bot or Talking avatar).ab,ti.

11 (Text-based healthcare chatbot or Text based dialogue system or Text-based synchronous chat or Speech recognition software or Voice recognition software).ab,ti.

12 (Virtual assistant or Virtual agent or Virtual advisor or Virtual conversational agent or Virtual coach or Virtual online assistant or Virtual personal assistant).ab,ti.

13 1 or 2 or 3 or 4 or 5 or 6 or 7 or 8 or 9 or 10 or 11 or 12

**Search Strategy on PsychINFO**

S1

TI ( “Artificially intelligent chatbot” or “Artificially intelligent chat agent” or “Automated virtual agent” or “Automated

chat agent” or “Artificial intelligence chatbot” or “Artificial conversational entity” or “AI agent” ) OR AB ( “Artificially

intelligent chatbot” or “Artificially intelligent chat agent” or “Automated virtual agent” or “Automated chat agent” or

“Artificial intelligence chatbot” or “Artificial conversational entity” or “AI agent” )

S2

TI ( “Chatterbox” or “Chatbot” or “Chatterbot” or “Chat assistant” or “chat bot” or “chat-bot” or “chatter bot” ) OR AB (

“Chatterbox” or “Chatbot” or “Chatterbot” or “Chat assistant” or “chat bot” or “chat-bot” or “chatter bot” )

S3

TI ( “Conversational agent” or “Conversational system” or “Conversational assistant” or “Conversational User

Interface” or “Conversational Personal Assistant” or “Conversational interface” or “Conversational avatar” or

“Conversational computer” or “Conversational humanoid” or “Conversational Character” or “Conversational bot” or

“Conversational AI” ) OR AB ( “Conversational agent” or “Conversational system” or “Conversational assistant” or

“Conversational User Interface” or “Conversational Perso Personal Assistant or Conversational interface or Conversational avatar or Conversational computer or Conversational humanoid or Conversational Character or Conversational bot or Conversational AI

S4

TI ( “Dialog system” or “Dialogue system” ) OR AB ( “Dialog system” or “Dialogue system” )

S5

TI ( “Embodied agent” or “Embodied conversational agent” ) OR AB ( “Embodied agent” or “Embodied

conversational agent” )

S6

TI ( “Interactive online character” or “Interactive virtual agent” or “Interactive agent” or “Interactive conversational

assistant” or “Intelligent virtual agent” or “Intelligent virtual assistant” or “Intelligent conversational assistant” or

“Intelligent conversational avatar” or Intelligent agent” or “Intelligent assistant” ) OR AB ( “Interactive online character” or

“Interactive virtual agent” or “Interactive agent” or “Interactive conversational assistant” or “Intelligent agent” or “Intelligent assistant”)

S7

TI ( “Smartbot” or “Smart bot” or “Smart virtual assistant” ) OR AB ( “Smartbot” or “Smart bot” or “Smart virtual

assistant” )

S8

TI ( “Online chat agent” or “Sociable agent” or “Relational agent” ) OR AB ( “Online chat agent” or “Sociable agent”

or “Relational agent” )

S9

TI ( “Talk bot” or “Talking avatar” ) OR AB ( “Talk bot” or “Talking avatar” )

S10

TI ( “Text-based healthcare chatbot” or “Text based dialogue system” or “Text-based synchronous chat” or “Speech

recognition software” or “Voice recognition software” ) OR AB ( “Text-based healthcare chatbot” or “Text based dialogue

system” or “Text-based synchronous chat” or “Speech recognition software” or “Voice recognition software” )

S11

TI ( “Virtual assistant” or “Virtual agent” or “Virtual advisor” or “Virtual conversational agent” or “Virtual coach” or

“Virtual online assistant” or “Virtual personal assistant” ) OR AB ( “Virtual assistant” or “Virtual agent” or “Virtual advisor”

or “Virtual conversational agent” or “Virtual coach” or “Virtual online assistant” or “Virtual personal assistant” )

S12

S1 OR S2 OR S3 OR S4 OR S5 OR S6 OR S7 OR S8 OR S9 OR S10 OR S11

**Search Strategy for Web of Science**

#1

(TI=(“Artificially intelligent chatbot” or “Artificially intelligent chat agent” or “Automated virtual agent” or “Automated chat agent” or “Artificial intelligence chatbot” or “Artificial conversational entity” or “AI agent”)) OR AB=(“Artificially intelligent chatbot” or “Artificially intelligent chat agent” or ” or “Automated virtual agent” or “Automated chat agent” or “Artificial intelligence chatbot” or “Artificial conversational entity” or “AI agent”)

#2

(TI=(“Chatterbox” or “Chatbot” or “Chatterbot” or “Chat assistant” or “chat bot” or “chat-bot” or “chatter bot”)) OR AB=(“Chatterbox” or “Chatbot” or “Chatterbot” or “Chat assistant” or “chat bot” or “chat-bot” or “chatter bot”))

#3

(TI=(“Conversational agent” or “Conversational system” or “Conversational assistant” or “Conversational User Interface” or “Conversational Personal Assistant” or “Conversational

interface” or “Conversational avatar” or “Conversational computer” or “Conversational humanoid” or “Conversational Character” or “Conversational bot” or Conversational AI”)

#4

(TI=(“Dialog system” or “Dialogue system” )) OR AB=(“Dialog

system” or “Dialogue system” )

#5

(TI=(“Embodied agent” or “Embodied conversational agent” ))

AND AB=(“Embodied agent” or “Embodied conversational agent” )

#6

(TI=(“Interactive online character” or “Interactive virtual agent”

or “Interactive agent” or “Interactive conversational assistant”

or “Intelligent virtual agent” or “Intelligent virtual assistant” or

“Intelligent conversational assistant” or “Intelligent

conversational avatar” or “Intelligent agent” or “Intelligent assistant”) AND AB=(“Interactive online character” or “Interactive virtual agent”

or “Interactive agent” or “Interactive conversational assistant”

or “Intelligent virtual agent” or “Intelligent virtual assistant” or

“Intelligent conversational assistant” or “Intelligent

conversational avatar” or “Intelligent agent” or “Intelligent assistant”)

#7

(TI=(“Smartbot” or “Smart bot” or “Smart virtual assistant” )) OR

AB=(“Smartbot” or “Smart bot” or “Smart virtual assistant” )

#8

(TI=(“Online chat agent” or “Sociable agent” or “Relational agent”

)) OR AB=(“Online chat agent” or “Sociable agent” or “Relational

agent” )

#9

(TI=(“Talk bot” or “Talking avatar” )) OR AB=(“Talk bot” or

“Talking avatar” )

#10

(TI=(“Text-based healthcare chatbot” or “Text based dialogue

system” or “Text-based synchronous chat” or “Speech

recognition software” or “Voice recognition software” )) OR AB=(“Text-based healthcare chatbot” or “Text based dialogue

system” or “Text-based synchronous chat” or “Speech

recognition software” or “Voice recognition software” )

#11

(TI=(“Virtual assistant” or “Virtual agent” or “Virtual advisor”

or “Virtual conversational agent” or “Virtual coach” or “Virtual

online assistant” or “Virtual personal assistant” )) OR AB=(“Virtual assistant” or “Virtual agent” or “Virtual advisor”

or “Virtual conversational agent” or “Virtual coach” or “Virtual

online assistant” or “Virtual personal assistant” )

#12

#11 OR #10 OR #9 OR #8 OR #7 OR #6 OR #5 OR #4 OR #3 OR #2 OR

#1

**Search strategy for Google Search**

Artificially intelligent chatbot OR Artificially intelligent chat agent OR Automated virtual agent OR Automated chat agent OR Artificial intelligence chatbot OR Artificial conversational entity OR AI agent OR Chatterbox OR Chatbot OR Chatterbot OR Chat assistant OR chat bot OR chat-bot OR chatter bot OR Conversational agent OR Conversational system OR Conversational assistant OR Conversational User Interface OR Conversational Personal Assistant OR Conversational interface OR Conversational avatar OR Conversational computer OR Conversational humanoid OR Conversational Character OR Conversational bot OR Conversational AI OR Dialog system OR Dialogue system OR Embodied agent OR Embodied conversational agent OR Interactive online character OR Interactive virtual agent OR Interactive agent OR Interactive conversational assistant OR Intelligent virtual agent OR Intelligent virtual assistant OR Intelligent conversational assistant OR Intelligent conversational avatar OR Intelligent agent OR Intelligent assistant OR Smartbot OR Smart bot OR Smart virtual assistant OR Online chat agent OR Sociable agent OR Relational agent OR Talk bot OR Talking avatar OR Text-based healthcare chatbot OR Text based dialogue system OR Text-based synchronous chat OR Speech recognition software OR Voice recognition software OR Virtual assistant OR Virtual agent OR Virtual advisor OR Virtual conversational agent OR Virtual coach OR Virtual online assistant OR Virtual personal assistant
