# Supplementary material for: Evaluating Conversational Agents for Mental Health: Scoping Review of Outcomes and Outcome Measurement Instruments
Source: J Med Internet Res. 2023 Apr 19;25:e44548. doi: 10.2196/44548 (PMC10157460; doi:10.2196/44548)
Supplement: Multimedia Appendix 3 [file jmir_v25i1e44548_app3.docx]

Multimedia Appendix 3: Selected definitions for Core Outcome Measures in Effectiveness Trials (COMET)’s medical research outcome taxonomy

| Core Area | Outcome domain | Definition/Explanation |
| --- | --- | --- |
| **Physiological/ clinical** | **Physiological/clinical**  21. Psychiatric outcomes | Psychiatric outcomes include all those relating to mental health conditions and associated behaviours (e.g. addictions and behavioural problems) |
| **Life impact** | 25. Physical functioning | Impact of disease/condition on physical activities of daily living (for example, ability to walk, independence, self-care, performance status, disability index, motor skills, sexual dysfunction, health behaviour and management) |
|  | 26. Social functioning | Impact of disease/condition on social functioning (e.g. ability to socialize, behaviour within society, communication, psychosocial development, aggression, recidivism, participation) |
|  | 27. Role functioning | Impact of disease/condition on role (e.g. ability to care for children, work status |
|  | 28. Emotional functioning/ wellbeing | impact of disease/condition on emotions or overall wellbeing (e.g. ability to cope, worry, frustration, confidence, perceptions regarding body image and appearance, psychological status, stigma, life satisfaction, meaning and purpose, positive affect, self-esteem, self-perception and self-efficacy) |
|  | 29. Cognitive functioning | Impact of disease/condition on cognitive functioning (e.g. memory lapse, lack of concentration, attention); outcomes relating to knowledge, attitudes, and beliefs (e.g. learning and applying knowledge, spiritual beliefs, health beliefs/knowledge) |
|  | 30. Global quality of life | Includes only implicit composite outcomes measuring global quality of life |
|  | 31. Perceived health status | Subjective rating by the affected individual of their relative level of health |
|  | 32. Delivery of care | Includes outcomes relating to the delivery of care, including   - Adherence/compliance - Patience preference - Tolerability/acceptability of intervention - Withdrawal from intervention (e.g. time to treatment failure, reason for stopping therapy) - appropriateness of intervention - accessibility, quality and adequacy of intervention - patient/carer satisfaction (emotional rather than financial burden) - process, implementation and service outcomes (e.g. overall health system performance and the impact of service provision on the users of services) |
|  | 33. Personal circumstances | Outcomes relating to patients finances, home, and environment |
| **Adverse events** | 38. Adverse events/effects | Includes outcomes broadly labelled as some form of unintended consequence of the intervention (e.g. adverse events/effects, adverse reactions, safety, harm, negative effects, toxicity, complications, sequelae). Specifically named adverse events should be classified within the appropriate taxonomy domain above with an additional level of categorisation which identifies that this outcome is being considered as an adverse event. |

Please see Dodd et al. (2018) [15] for the full taxonomy.
