# Supplementary material for: Evaluating Conversational Agents for Mental Health: Scoping Review of Outcomes and Outcome Measurement Instruments
Source: J Med Internet Res. 2023 Apr 19;25:e44548. doi: 10.2196/44548 (PMC10157460; doi:10.2196/44548)
Supplement: Multimedia Appendix 5 [file jmir_v25i1e44548_app5.docx]

**Multimedia Appendix 5.** Outcome measures included in more than one study.

| Outcome Measurement Name | Studies included |
| --- | --- |
| Patient Health Questionnaire-9 (PHQ-9)^a^ | [3,4,26,32,34,43,44] |
| PANAS - Positive Affect Negative Affect Schedule^b,c^ | [3,26,30,32,48] |
| GAD-7 - Generalized Anxiety Disorder Scale | [3,4,26,32] |
| State-Trait Anxiety Inventory (STAI) | [24,41,43,48] |
| Perceived Stress Scale (PSS)^d,e^ | [35,36,44,52] |
| Depression, Anxiety, and Stress Scales 21 (DASS-21) | [27,28,51] |
| Helpfulness Questionnaire | [27,28,51] |
| Problem-related distress Questionnaire | [27,28,51] |
| Problem resolution Questionnaire | [27,28,51] |
| Working Alliance Inventory (WAI)^f^ | [4,13,43] |
| Quick Inventory of Depressive Symptoms-Self-Report (QIDS)^g^ | [35,50] |
| System Usability Scale (SUS) | [27,37] |
| Technology Acceptance Model (TAM) | [40,41] |
| Rosenberg Self-Esteem Scale | [41,42] |
| Toronto Mindfulness Scale | [24,48] |
| Jackson’s Flow State Scale | [24,48] |

^a^includes shortened version of PHQ-9 (PHQ-8); ^b^includes German translation of PANAS (PANAS-G); ^c^includes shortened version of PANAS (PANAS-SF); ^d^includes Korean translation of PSS; ^e^include Chinese translation of PSS; ^f^includes shortened version of WAI; ^g^includes Korean translation of QIDS.
