# Supplementary material for: Evaluating Conversational Agents for Mental Health: Scoping Review of Outcomes and Outcome Measurement Instruments
Source: J Med Internet Res. 2023 Apr 19;25:e44548. doi: 10.2196/44548 (PMC10157460; doi:10.2196/44548)
Supplement: Multimedia Appendix 6 [file jmir_v25i1e44548_app6.docx]

**Multimedia Appendix 6.** System log data and definitions provided by the studies.

| **ID** | **Definition from included studies** | **Categories*** | |
| --- | --- | --- | --- |
| Bennion-2020 | time spent measured in minutes | total duration of use | |
| Burton-2016 | The median number of times that Help4Mood was used was 10.5 (twice a week or more) and the median total duration was 134 minutes. Two participants appeared to have used the system for only one or two days, three used it on 3—7 days (casual users), and six used Help4Mood at least twice a week (10 times or more, regular users). | program use by day or week | |
| Burton-2016 | time spent measured in minutes and median of time spent across sessions | total duration of use | |
| Fitzpatrick-2017 | An interaction was deemed to have taken place if mood and context data were recorded. | assessment of active use | |
| Fitzpatrick-2017 | time spent measured in minutes and seconds | total duration of use | |
| Fulmer-2018 | number of messages exchanged between Tess and the participants per group and in total | interaction with CA | |
| Gaffney-2014 | average time spent in minutes | total duration of use | |
| Gaffney-2014 | participants conversation log | assessment of active use | |
| Greer-2019 | engagement with the bot lasting at least two user inputs within 5 minutes and a break no longer than 5 minutes | assessment of active use | |
| Greer-2019 | the count of all sessions for an individual user | total number of sessions | |
| Greer-2019 | total time from the start to the end of a session summed across all sessions | total duration of use | |
| Greer-2019 | count of any session that included, at minimum, a completion of the 6-item emotion rating | total number of sessions | |
| Greer-2019 | average duration in minutes of any session that included, at minimum, a completion of the 6-item emotion rating | average duration of session | |
| Hudlika-2013 | higher frequency of answering the surveys | adherence to usage instruction | |
| Hunt-2021 | number of modules completed | completion of structured module | |
| Jang-2021 | One check-in was defined as access being established at least 10 min from the previous activity interruption. | assessment of active use | |
| Jang-2021 | Total use time in seconds, minutes, hour, day | total duration of use | |
| Jang-2021 | number of module used e.g. daily check-up, psychoeducation session, mindfulness, breath exercise & muscle relaxation | use of specific program features |  |
| Ly-2017 | as completing at least 14 reflections over the course of 14 days, as well as not being inactive for 7 or more days in a row | assessment of active use |  |
| Ly-2017 | how many times per day participants opened the app to have a conversation with Shim (1 conversation contained in average 3 reflections) | assessment of active use |  |
| Oh-2020 | checked in app | program use by day or week |  |
| Oh-2020 | total time spent | total duration of use |  |
| Prochaska-2021 | days of app use | program use by day or week |  |
| Prochaska-2021 | number of messages sent | interaction with CA |  |
| Prochaska-2021 | number of modules/lessons completed | completion of structured module |  |
| **ID** | **Definition from included studies** | **Categories^a^** |  |
| So-2020 | any reaction by participants to LINE messages on any day | interaction with CA |  |
| So-2020 | number of days using GAMBOT | total duration of use |  |
| Bickmore-2010 | total session, | total number of sessions |  |
| Bickmore-2010 | average activity usage | average duration of session |  |
| Bickmore-2010 | module used, | use of specific program features |  |
| Huang-2015 | After the 1-month user study, we obtained: 1) 62 conversations | interaction with CA |  |
| Huang-2015 | After the 1-month user study, we obtained: … 1063 chatting sentences | interaction with CA |  |
| Huang-2015 | After the 1-month user study, we obtained: ... 6.2 conversations for each user and 17.2 utterances for each conversation in average | interaction with CA |  |
| Huang-2015 | After the 1-month user study, we obtained: ... 17.2 utterances for each conversation in average | interaction with CA |  |
| Narain-2020 | the research team analyzed key features of messages written on the Sunny platform. Figure 4 presents the identified features and provides examples for each. | interaction with CA |  |
| Narain-2020 | on average across all three groups, participants sent more messages than they received - typically a user received one message and then sent messages to 2-3 participants in the group, though they were only required to send one message per day as part of the study activities | interaction with CA |  |
| Ring-2018b | To​ ​assess​ ​the​ ​efficacy​ ​of​ ​the​ ​system,​ ​the​ ​user’s​ ​system​ ​usage​ ​(the​ ​number​ ​of​ ​sessions​ ​...) | total number of sessions |  |
| Ring-2018b | To​ ​assess​ ​the​ ​efficacy​ ​of​ ​the​ ​system,​ ​the​ ​user’s​ ​system​ ​usage​ ​(... ​the session​ ​duration) | average duration of session |  |
| Shamekhi-2016 | Based on analysis of log files from the tablet computers, patients spent an average total of 90 min (range 0–294, SD 80.2) interacting with the virtual agent at home over the nine weeks of the intervention. | total duration of use |  |
| Shamekhi-2017 | The analysis of log files from tablet computers suggests that intervention participants interacted with Gabby an average of 105 min … over the first 9 weeks they had the system. | total duration of use |  |
| Shamekhi-2017 | The analysis of log files from tablet computers suggests that intervention participants interacted with Gabby … with the average number of 8.8 logins, over the first 9 weeks they had the system. | total number of sessions |  |
| Sidner-2018 | total session | total number of sessions |  |
| Sidner-2018 | total min used, days used | total duration of use |  |
| Sidner-2018 | module used | use of specific program features |  |

Note. ^a^Categories based on the systematic review by Molloy and Anderson [10] on engagement with mobile health interventions.
